# Rapid HIV disease progression following superinfection in an HLA-B*27:05/B*57:01-positive transmission recipient

**DOI:** 10.1186/s12977-018-0390-9

**Published:** 2018-01-16

**Authors:** Jacqui Brener, Astrid Gall, Jacob Hurst, Rebecca Batorsky, Nora Lavandier, Fabian Chen, Anne Edwards, Chrissy Bolton, Reena Dsouza, Todd Allen, Oliver G. Pybus, Paul Kellam, Philippa C. Matthews, Philip J. R. Goulder

**Affiliations:** 1 Department of Paediatrics, University of Oxford, Oxford, UK; 2 Department of Veterinary Medicine, University of Cambridge, Cambridge, UK; 3 Wellcome Trust Sanger Institute, Wellcome Trust Genome Campus, Hinxton, Cambridge, UK; 4 Nuffield Department of Medicine, University of Oxford, Oxford, UK; 5 Ragon Institute of MGH, MIT and Harvard, Boston, MA USA; 6 Department of Sexual Health, Royal Berkshire Hospital, Reading, UK; 7 Department of GU Medicine, The Churchill Hospital, Oxford University NHS Foundation Trust, Oxford, UK; 8 Department of Zoology, University of Oxford, Oxford, UK; 9 Division of Infection and Immunity, University College London, Gower Street, London, UK

**Keywords:** HIV-1, HLA, CTL response, Transmission pair, Superinfection, Ultra-deep sequencing

## Abstract

**Background:**

The factors determining differential HIV disease outcome among individuals expressing protective HLA alleles such as HLA-B*27:05 and HLA-B*57:01 remain unknown. We here analyse two HIV-infected subjects expressing both HLA-B*27:05 and HLA-B*57:01. One subject maintained low-to-undetectable viral loads for more than a decade of follow up. The other progressed to AIDS in < 3 years.

**Results:**

The rapid progressor was the recipient within a known transmission pair, enabling virus sequences to be tracked from transmission. Progression was associated with a 12% Gag sequence change and 26% Nef sequence change at the amino acid level within 2 years. Although next generation sequencing from early timepoints indicated that multiple CD8+ cytotoxic T lymphocyte (CTL) escape mutants were being selected prior to superinfection, < 4% of the amino acid changes arising from superinfection could be ascribed to CTL escape. Analysis of an HLA-B*27:05/B*57:01 non-progressor, in contrast, demonstrated minimal virus sequence diversification (1.1% Gag amino acid sequence change over 10 years), and dominant HIV-specific CTL responses previously shown to be effective in control of viraemia were maintained. Clonal sequencing demonstrated that escape variants were generated within the non-progressor, but in many cases were not selected. In the rapid progressor, progression occurred despite substantial reductions in viral replicative capacity (VRC), and non-progression in the elite controller despite relatively high VRC.

**Conclusions:**

These data are consistent with previous studies demonstrating rapid progression in association with superinfection and that rapid disease progression can occur despite the relatively the low VRC that is typically observed in the setting of multiple CTL escape mutants.

**Electronic supplementary material:**

The online version of this article (10.1186/s12977-018-0390-9) contains supplementary material, which is available to authorized users.

## Background

Outcome in HIV infection is strongly influenced by the particular HLA alleles that are expressed, the most protective of which are HLA-B*27:05 and HLA-B*57:01 [1, 2]. The mechanism by which these HLA alleles confer protection is related to the presentation of a broad array of epitopes that enable CD8+ cytotoxic T lymphocytes (CTL) to recognise and kill HIV-infected target cells [2, 3]. As HIV-specific CTL are likely to play a critical role in strategies designed to eradicate viral reservoirs [4], it remains important to define what features affect the ability of CTL to be effective in killing virus-infected cells. We here contrast HIV disease outcomes in two HIV-infected subjects expressing both HLA-B*27:05 and HLA-B*57:01. One subject is a typical HIV non-progressor, maintaining viral loads below the level of detection for more than a decade of follow up. The other subject initially presented the characteristic phenotype of an HLA-B*27:05/57:01-positive HIV-infected subject, with a low viral load (65 copies/ml) and high absolute CD4+ T cell count (950 cells/mm^3^), but within 2.7 years had progressed rapidly to AIDS (CD4+ T cell count < 200 cells/mm^3^).

## Methods

### Study subjects

The adult Caucasian transmission pair R096 (HLA-A*02:01/A*11:01 B*13:02/B*51:01 C*03:03 C*06:02) and R097 (HLA-A*11:01/A*24:01 B*27:05/B*57:01 C*02:02 C*06:02) and non-progressor RI088 (HLA-A*01:01/A*03:01 B*27:05/B*57:01 C*02:02 C*06:02) were recruited from the Thames Valley Cohort, UK, previously described [5].

### DNA and RNA extraction

Proviral and genomic DNA were extracted for proviral sequencing and HLA-typing respectively, using PureGene reagents according to the manufacturers instructions. Viral plasma RNA was extracted using the Qiamp Viral RNA Mini Kit (Qiagen, UK) according to the manufacturers instructions. For RNA extraction for ultra-deep sequencing, minor modifications to the manufacturers protocol were made, as previously described [6].

### HLA-typing

Four digit HLA Class I typing was performed at the CLIA/ASHI accredited laboratory of William Hildebrand, PhD, D (ABHI), University of Oklahoma Health Sciences Center. Locus specific PCR amplification of exons 2 and 3 and heterozygous sequencing were performed from genomic DNA. Ambiguities were resolved by homozygous sequencing [7].

### IFN-γ ELISpot assays

Peripheral blood mononuclear cells were tested for responses to 410 18mer overlapping peptides spanning the B clade HIV proteome as previously described [8] using pools of 11–12 peptides followed by confirmatory assays using individual 18mer peptides.

### Sanger sequencing of proviral DNA

The *gag* gene was amplified from proviral DNA using a nested touchdown PCR with BioTaq DNA polymerase (Bioline, UK) using the following primers: 5′-CTCTAGCAGTGGCGCCCGAA-3′ and 5′-TCCTTTCCACATTTCCAACAGCC-3′ for the first round PCR and 5′-ACTCGGCTTGCTGAAGTGC-3′ and 5′ CAATTTCTGGCTATGTGCCC-3′ for the second round PCR. Twenty cycles of denaturation at 94 °C for 15 s, annealing at 60 °C for 30 s and elongation at 72 °C for 1 min were performed followed by another 20 cycles with an annealing temperature of 57 °C. Purified PCR product was used to prepare sequencing templates using BigDye Terminator v3.1 reaction mix (Applied Biosystems, UK) and sequenced on an ABI 3730xl DNA Analyzer (Applied Biosystems, UK) by the Department of Zoology Sequencing Facility, University of Oxford. For clonal sequencing, purified PCR product was cloned into TOPO vectors using Zero Blunt TOPO PCR Cloning Kit (Invitrogen, UK) and used to transform chemically competent One Shot TOP10 *E. coli* cells (Invitrogen, UK) according to the manufacturers instructions. Where possible, clones were selected from two independent PCRs to avoid PCR amplification bias. Selected colonies were cultured overnight in LB broth before proceeding with mini-prep plasmid DNA extraction using Montage 96-well plasmid preparation kits (Millipore, US) according to the manufacturers instructions. Plasmid DNA was used for sequencing template preparation as described above.

### Ultra-deep sequencing de novo assembly of consensus sequences and minor variant haplotype analysis

The full-length HIV genome was amplified in four fragments from plasma RNA using Superscript III One-Step RT PCR Kit with Platinum Taq High Fidelity enzyme (Invitrogen, UK) as previously described [9]. Sequencing of pooled amplicons was performed using Illumina MiSeq 250 bp paired-end technology. Quality control of reads was performed using QUASR (http://sourceforge.net/projects/quasr/) as previously described [6, 10, 11]. A de novo assembly was constructed using SPAdes version 2.4.0 [12] and a consensus sequence was generated using Abacas version 1.3.1 and MUMmer version 3.2 [13]. Haplotypes in the epitope regions were determined using *Vprofiler* [12] by selecting reads that span the epitope region and which contain only accepted variants.

### Phylogenetic analysis and recombination detection

Maximum likelihood phylogenetic trees were constructed using Mega 6.06 software under the General Time Reversible model of nucleotide substitution as determined by jModelTest version 0.1.1 [14] with 1000 bootstrap replicates and viewed using FigTree v1.4.0 software. Recombination analysis was performed using RDP 4.46 (Recombination Detection Programme) [15] and SimPlot 3.5.1 [16].

### Generation of recombinant *gag*-*protease* HIV virions and viral replicative capacity assays (VRC)

Recombinant viral vectors expressing autologous-*gag*-*protease* sequences from HLA-B*27:05/HLA-B*57:01+ individuals were constructed by transfecting *gag*-*protease* amplicons into the *Gag*-*Pro* deleted HIV backbone ΔGag-Pro NL4-3 as previously described [17, 18]*. Gag*-*protease* was amplified by nested PCR from plasma RNA using previously published primers [18]. The first round amplification was performed using Superscript III One-Step RT PCR Kit with Platinum Taq High Fidelity enzyme (Invitrogen, UK) followed by second round PCR using High Fidelity Platinum (Invitrogen, UK) according to the manufacturers’ instructions. PCR product was purified using QIAquick PCR Purification Kit (Qiagen, UK) and sequenced by Sanger sequencing to confirm accurate amplification of the patient-derived *gag*-*protease* sequence.

The ΔGag-Pro NL4-3 was produced by introducing BstEII restriction enzyme sites on either side of the *gag*-*protease*-gene in NL4-3, followed by BstEII digest to delete *gag*-*protease* and allow self ligation of the plasmid [18]. For transfection, the plasmid was linearised by BstEII digest at 60 °C for 2 h. For each transfection, 2.5 μg of purified *gag*-*protease* amplicon and 10 μg of linearised ΔGag-Pro NL4-3 were co-transfected into 2 million CEM-GXR reporter cells [17, 19] by electroporation (300 V, 500 μF capacitance and infinite resistance). Recombinant viral cultures were expanded over 1 month and viral spread measure by GFP expression detected by flow cytometry. Viral supernatant was collected when > 30% of live cells were infected. Viral RNA extraction and *gag*-*protease* sequencing confirmed the correct sequence amplification of viral stocks. Viral titres were determined by infection of 1 million CEM-GXR cells with a standardized volume of viral supernatant and examining GFP expression after 48 h.

 Viral replicative capacity assays (VRC): CEM-GXR cells were infected in triplicate at a low multiplicity of infection (0.025% GFP+ cells at day 2). GFP expression was monitored daily by flow cytometry over 12 days.

### Logistic curve modelling of viral replicative capacity data

Viral growth curves were modeled on the logistic curve function:

$$\begin{aligned} y & = m\left( {x,\theta } \right) + \varepsilon \\ & = \theta _{1} /1 + \exp \left( { - \theta _{2} + \theta _{3} x} \right) \\ \end{aligned}$$

where θ_1_ represents the asymptotic y point, θ_2_ the midpoint, and θ_3_ the scaling factor (rate of change) of the curve. Curves were fitted using the nonlinear regression nls function in R version 3.2.1, plotting the best fit logistic growth curve. To assess whether each parameter was statistically different when comparing curves, models were built where one of the parameters was fixed while the others were allowed to vary. A model where all parameters were allowed to vary was then fitted and a statistical comparisons of parameters was made using ANOVA. The parameters used for the curves in Fig. 4 are shown in Additional file 1: Table 1, together with the statistics for the overall fit and for the separate parameters.

### Sequence accession numbers

Sequence data has been deposited in Genbank under the accession numbers MF039091-MF039203.

## Results

### Rapid progression to AIDS in a transmission recipient expressing HLA-B*27:05/B*57:01

**Fig. 1:**
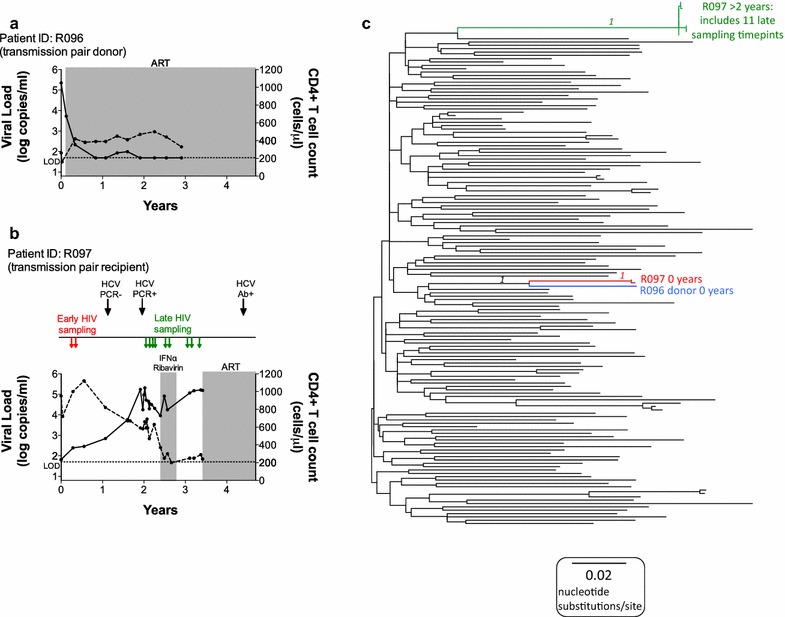
Plasma HIV RNA viral load and CD4+ T cell counts for HLA-B*27:05/57:01+ subject R097 and his transmission partner R096 and phylogenetic analysis of HIV sequences showing epidemiological linkage and sequence diversity in transmission pair R096/R097. **a** Transmission pair donor, R096. **b** Transmission pair recipient, R097. ‘Early’ and ‘late’ time points of sampling for sequencing are shown (see **c**). ‘Time 0’ represents the time of diagnosis of recipient R097. The horizontal dotted line represents the limit of detection (LOD) of the viral load assay (40 HIV RNA copies/ml). Grey shading indicates the period during which the subject received antiretroviral (ART) therapy. **c** Maximum likelihood phylogenetic tree of 1091 bp alignment of RNA (ultra-deep consensus) sequences across the Gag p17 and p24 genes. The donor R096 sequence is shown in blue. Early and late sequencing time points are shown in **a**. The early R097 sequences are shown in red (n = 2). The late (post-2 year) sequences for R097 are shown in green (n = 11). The early and late sequences form two distinct clusters indicating considerable intra-host sequence diversification over time. 143 B clade reference sequences from the US and UK collected between 2003 and 2011 from the Los Alamos database (http://www.hiv.lanl.gov/) are shown in black. Bootstrap values for the R097 sequence clusters based on 1000 bootstrap replicates are shown in italics

The starting point for this study was the MSM transmission pair R096/R097 (Fig. 1a, b). The transmission recipient, R097, expressed both HLA-B*27:05 and HLA-B*57:01, both of which are strongly associated with slow progression to AIDS. The viral load in R097 at diagnosis (time 0) was 65 HIV RNA copies/ml plasma and the absolute CD4+ T cell count was 950 cells/mm^3^, reflecting successful immune control of HIV. Over the next 2–3 years, however, despite the co-expression of HLA-B*27:05 and HLA-B*57:01, progression to AIDS (absolute CD4+ T cell < 200 cells/mm^3^) occurred rapidly, in association with viral loads increasing to > 10^5^ copies/ml. The donor R096 at time 0 had a viral load of 221,839 copies/ml and an absolute CD4+ T cell count of 260 cells/mm^3^ and initiated antiretroviral therapy (ART) at this time.

To investigate why disease had progressed so rapidly in R097, and to seek evidence to support the clinical history suggesting transmission from R096 to R097, we ultra-deep sequenced the virus in both individuals (Fig. 1c). Tight phylogenetic clustering of the viral sequences at ‘time 0’ in R096 and R097 strongly supported the notion that R096/R097 were a transmission pair. Viral sequences from R097 post-progression (2.0–3.4 years after ‘time 0’) clustered separately from the time 0 sequences. Gag sequences in R097 differed by 12% at the amino acid level in the 2 years from ‘time 0’ and Nef sequences by 26% at the amino acid level over this short time. These changes occurred in the context of a 12.5% nucleotide change across the full-length genome.

The direction of transmission from R096 to R097 was supported by the presence of virus encoding escape mutants in five HLA-B*51:01-restricted CTL epitopes in both subjects (Table 1). Since HLA-B*51:01 is expressed by R096 and not R097, this is consistent with the variant being selected initially in R096 and being transmitted to R097. In four of the HLA-B*51:01-restricted epitopes, the variant we observed was the one most commonly seen in association with HLA-B*51:01, a so-called HLA footprint [for example, I135T within the epitope TAFTIPSI (RT 128-135)] [20–22]. In contrast, no HLA-B*57:01 or HLA-B*27:05-associated mutants were observed in the ‘time 0’ sequences in R096 and R097 (Table 2). One variant L268I was shared by R096 and R097 at ‘time 0’, which lies within the HLA-B*27:05-restricted epitope KRWIILGLNK (Gag 263-272). However, this mutant is an HLA-B*08:01-associated variant flanking the overlapping HLA-B*08:01 epitope EIYKRWII (Gag 260-267) [22] and thus is likely to have been originally selected in an HLA-B*08:01-positive subject prior to transmission to R096.

**Table 1:**
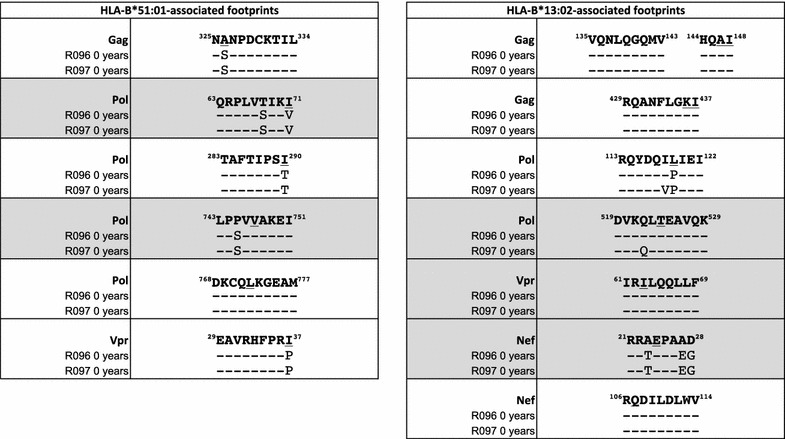
HLA-B*51:01 and HLA-B*13:02 footprints in the recipient R097 sequence

For each epitope the B clade consensus sequence is represented as the top line. The R096 (donor) sequence is represented on the middle line, and the R097 (recipient) sequence at time 0 on the bottom line. Polymorphisms that represent previously described HLA-associated footprints [22] underlined. Sequences where an HLA-associated footprint does not fall within a described epitope are shaded in grey

**Table 2:**
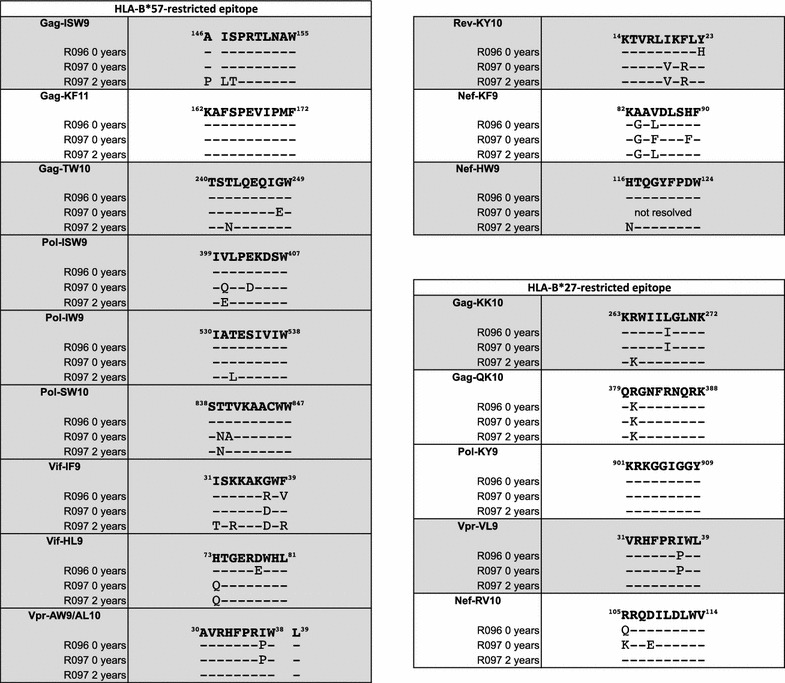
Donor R096 and recipient R097 viral sequences encoding HLA-B*27:05/B*57:01-restricted epitopes

Epitopes showing evidence of CTL driven evolution in R097 are highlighted in grey

### Mechanism of distinct phylogenetic clustering of R097 sequences following progression

Comparison of the viral sequences in R097 at ‘time 0’ and the cluster following progression 2.0–3.4 years later showed dramatic differences, prompting the hypothesis that R097 might have progressed as a consequence of HIV superinfection. To determine whether the dramatic sequence changes observed were likely to be the result of superinfection, or of multiple escape mutations being selected over a short period of time to give the appearance of superinfection, we first sought evidence of escape within HLA-B*27:05 and HLA-B*57:01-restricted epitopes selected between ‘time 0’ and 2.0 years later (Table 2). In patient R097 between ‘time 0’ and 2.0 years later, we examined 17 HLA-B*27:05/57:01 epitopes previously shown to contain well-characterised footprints [22], and found escape mutants in 12 of these (one mutation being contained in two overlapping HLA-B*27:05 and HLA-B*57:01 epitopes in Vpr). Additional mutants were observed that were selected between ‘time 0’ and 2.0 years later in HLA-B*27:05/57:01 restricted epitopes, but where HLA footprints have not been described (for example in the HLA-B*27:05 epitope GRRGWEALKY). Thus a proportion of the amino acid differences between the ‘time 0’ sequence and the viral sequences post progression can be explained by the selection of escape mutants in R097. However this did not distinguish rapid selection of escape mutants from the possibility of superinfection with an HIV variant carrying multiple HLA-B*27:05 and HLA-B*57:01-associated escape variants.

To examine this further, we analysed the timing of selection of escape mutants in R097 using ultra-deep sequencing (Table 3). At ‘time 0’ in R097, many of the escape variants that had reached fixation 2.0 years later were already present at low frequencies. For example, Gag-A146P was present at 19%, Gag-T242N at 4%, Gag-R264K at 3%, and Pol-T839N at 50% in R097 at ‘time 0’, and all were present at 99 or 100% 2.0 years later. Each of these variants were present in 0% of viruses in R096 at ‘time 0’. These data are consistent with the rapid increase in frequency of escape mutations in multiple epitopes restricted by HLA-B*27:05 and HLA-B*57:01 in the 2 years after ‘time 0’, but clearly selection of escape mutants had already been initiated by ‘time 0’.

**Table 3:**
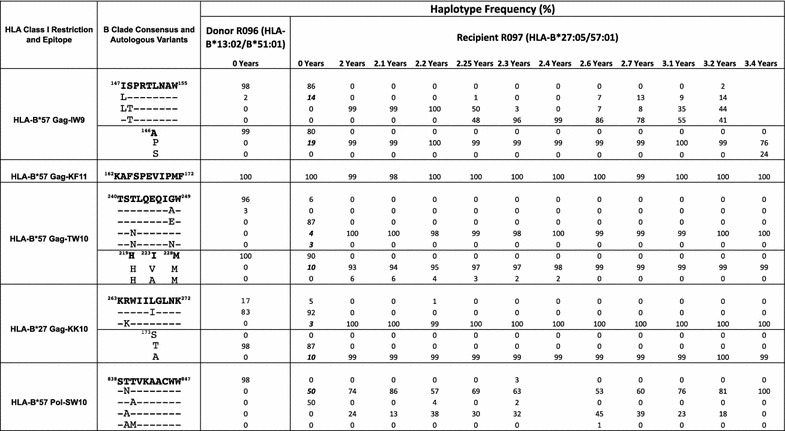
Ultra-deep RNA sequencing of HLA-B*57:01 and HLA-B*27:05-restricted epitopes and associated compensatory positions from R097 and his transmission partner R096

Depth of coverage ranges from 46 to 85,000 reads. Epitopes at which predictable footprints [22] are selected in the donor are shown. The early selection of escape and compensatory mutations in the minor variant populations are shown in bold italics

However, two features of the sequence data argue strongly in favour of superinfection having occurred in R097. First, some of the escape mutants selected at ‘time 0’ and not shared at the ‘time 2.0’ timepoint, and vice versa, are relatively rare. Examples here are A248E (87% at ‘time 0’ and 0% at ‘time 2.0’), which is a highly effective escape mutant [18], and S148T (0% at ‘time 0’ and 100% at ‘time 2.0’). Both of these mutants are within HLA-B*57:01-restricted CTL epitopes, but they are not selected sufficiently often to be included as HLA-B*57:01-associated ‘footprints’ [22]. In contrast, many of the HLA-B*27:05/B*57:01-associated escape mutants present both at ‘time 0’ and 0% at ‘time 2.0’, such as T242N and R264K, are commonly observed footprints for these HLA alleles [22], and could easily have arisen independently in the two viruses in different subjects expressing HLA-B*27:05/57:01. Second, the number of amino acid differences between the viruses at ‘time 0’ and at ‘time 2.0’ across the full-length proteomes numbered 810. Less than 3% of these amino acid changes (19/810) can be ascribed to escape arising within epitopes that are restricted by the HLA alleles expressed by R097. Thus the dramatic sequence changes observed in R097 between ‘time 0’ and 0% at ‘time 2.0’ cannot have been driven by CTL escape alone, but are consistent with HIV superinfection.

### HCV co-infection as an additional explanation for rapid immunological escape

Having established that HIV superinfection was likely to account for the rapid evolutionary changes in HIV sequences and disease progression in subject R097, we considered alternative explanations for the uncharacteristic speed of progression in an HLA B*27/B*57-positive individual. At the time of peak HIV viraemia (time 2.0 years) the recipient was diagnosed with hepatitis C virus (HCV) genotype 1a co-infection by PCR (viral load 4.03 log copies/ml), although his HCV antibody test remained negative for a further 2.5 years before he finally seroconverted. An HCV RNA test 10 months earlier (that is, 14 months after ‘time 0’) had been negative. He went on to receive treatment with IFNα/Ribavirin for 24 weeks, starting 5 months after the HCV diagnosis was made, although this did not result in clearance. Although HCV coinfection typically accelerates disease progression in HIV infection [23], in subject R097, as described above, escape mutants in multiple HIV-specific HLA-B*27:05- and HLA-B*57:01-restricted epitopes had already been selected, and the viral load was on an upward trajectory, more than a year prior to the HCV infection. Nonetheless, it is evident that HIV superinfection occurred either simultaneous with, or weeks-to-months before, HCV co-infection. In either case, HCV co-infected is likely to have accelerated the speed of progression to AIDS in this subject.

### Lack of sequence diversification in an HLA-B*27:05/57:01-positive non-progressor

**Fig. 2:**
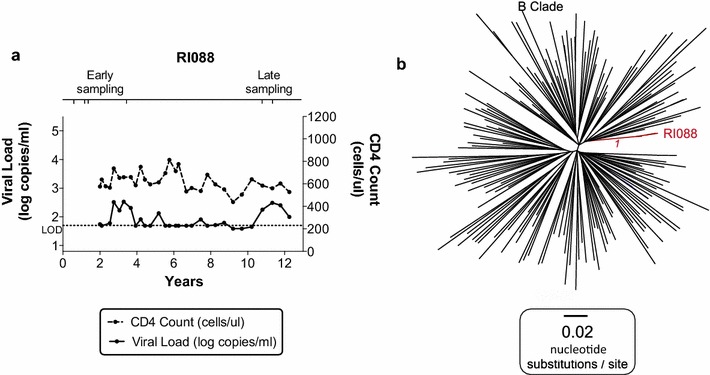
Clinical course of infection and phylogenetic analysis of viral sequences from HLA-B*27:05/57:01-positive controller RI088. **a** Plasma HIV viral load and CD4+ T cell count for HLA-B*27:05/B*57:01-positive recipient RI088. ‘Time 0’ represents the time of diagnosis. The horizontal dotted line represents the limit of detection (LOD) of the viral load assay (40 copies/ml). **b** Maximum likelihood phylogenetic tree of a 1089 bp alignment of the Gag gene sequenced from proviral DNA (consensus of clonal sequences shown in Table 4). Subject RI088 (n = 6) sequences spanning 10 years are shown in red. 180 B clade reference sequences from the US and UK collected between 2003 and 2011 from the Los Alamos database (http://www.hiv.lanl.gov/) are shown in black. Bootstrap values for the RI088 sequence cluster based on 1000 bootstrap replicates are shown in italics

**Fig. 3:**
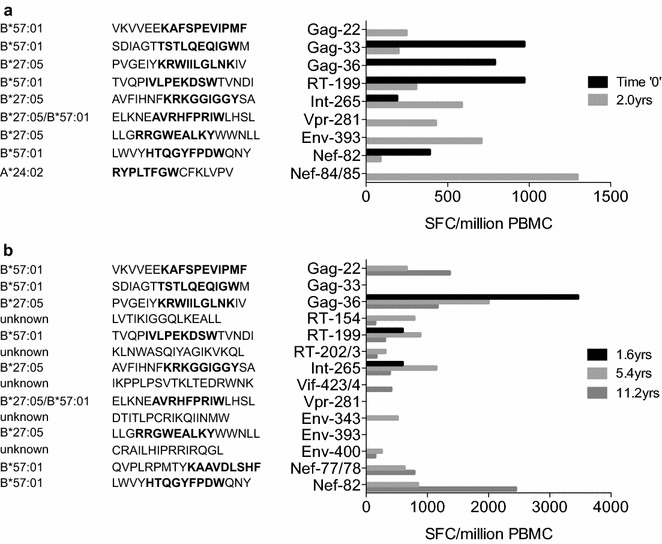
HIV-specific CTL responses in progressor R097 and in non-progressor RI088. EliSpot assays tested recognition of overlapping 18mer peptides, which together spanned the B clade proteome. The figure includes the full sequence of each 18mer peptide recognised and the HLA restriction where known, with known optimal epitopes shown in bold. **a** EliSpot responses detected in progressor R097 at time 0 (diagnosis) and 2.0 years later. Data shown at 2.0 years are the median of 3 assays undertaken at 1.96, 1.98 and 2.15 years after time 0. No responses were detected to the 18mer peptides containing the epitopes ISPRTLNAW or STTVKAACWW at either timepoint. **b** Responses detected in non-progressor RI088 at the times shown post diagnosis. Selected peptides that were strongly recognized in R097 but not in RI088 are included in panel B

To contrast the speed and extent of viral escape in the HLA-B*27:05/57:01-positive rapid progressor, R097, even before superinfection had occurred, with the sequence changes that might be expected in a non-progressor, we analysed *gag* viral sequences over a 10-year period in an ART-naive HLA-B*27:05/57:01-positive individual RI088, whose viral loads remained at < 1000 copies/ml, and at < 40 copies/ml for much of this period (Fig. 2). In this instance, viral sequence diversification was negligible with 1.1% difference in Gag at the amino acid level over 10 years. In contrast, we saw a 12% difference at the amino acid level in Gag over 2 years in the rapid progressor R097. Analysis of viral sequence at the clonal level in RI088 (Table 4, Additional file 2: Figure 1) shows that escape mutants were generated periodically but not always selected. For example, the most commonly selected variant, T242N, within the HLA-B*57:01 epitope TSTLQEQIGW (Gag 240–249), reached a frequency of 55% 1.4 years after diagnosis, but subsequently declined to 0% (at 3.4 years) before being selected later. Variants within KF11 and KK10 were observed transiently and at low frequencies. The relative lack of sequence change in the virus in RI088 is also reflected in persistence of CTL responses, such as to the HLA-B*27:05-restricted Gag epitope KK10 and the HLA-B*57:01-restricted Nef epitope HW9 (Fig. 3). In contrast, although these same responses are present at ‘time 0’ in R097, they declined in association with the selection of escape (Fig. 3).

**Table 4:**
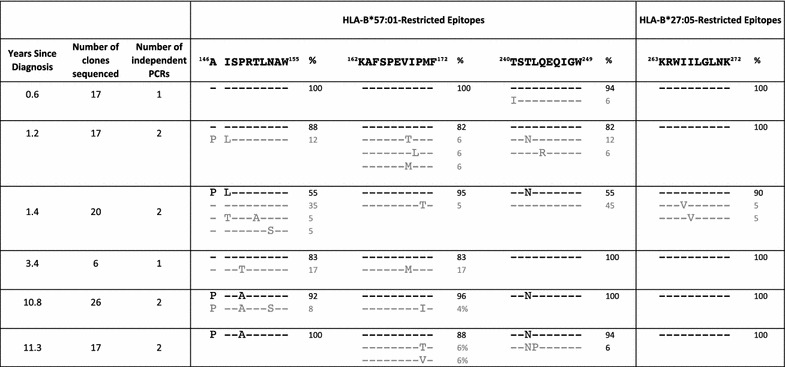
Clonal Sequencing of HIV proviral DNA at HLA‐B*57:01 and HLA-B*27:05-restricted Gag epitopes from controller RI088

The number of clones sequenced at each timepoint and number of independent PCR reactions from which PCR product was cloned are given. The percentage of clones with each sequence haplotype is shown. The most prevalent (consensus) epitope sequence is shown in black. Lower frequency variant sequences are shown in grey.

### Progression to AIDS in subject R097 associated with increasing viral replicative capacity

**Fig. 4:**
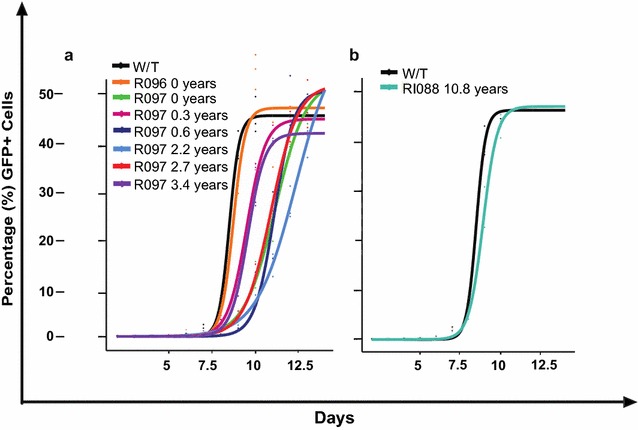
Viral replicative capacity (VRC) of recombinant viruses produced from autologous *gag*-*pro* from HLA-B*27:05/B*57:01-positive subjects. VRC of *gag*-*pro* chimeric virus derived from **a** longitudinal sampling of progressor R097 and his transmission partner R096; and **b** controller RI088. VRC is given by the percentage of infected (GFP-expressing) target cells

One of the mechanisms that has been proposed for HLA-B*27:05 and HLA-B*57:01-mediated protection against progression to HIV disease is the observation that the Gag escape mutations associated with these HLA molecules significantly reduce viral replicative capacity [5, 24–27]. Thus, even if the virus succeeds in evading the effective Gag-specific CTL responses restricted by HLA-B*27:05/57:01, the cost of reduced VRC facilitates maintained control of HIV through other immune responses from which the virus has not escaped. However, it is also well-described that reductions in viral replicative capacity resulting from selection of certain escape mutants are typically mitigated, to a varying degree, by selection of alternative escape mutants [28] and the co-selection of compensatory mutants [27, 29–31]. To investigate whether VRC was substantially reduced in the face of the selection of, or superinfection by a virus carrying, multiple Gag escape mutants in R097, we generated chimeric viruses comprising NL4-3 and the autologous *gag*-*pro* sequence from R097 (Fig. 4a). At ‘time 0’, when the viral load was low and the CD4+ T cell count was high, the VRC in R097 was substantially lower than both NL4-3 wildtype and ‘time 0’ R096 virus. However, during the course of progression, the VRC ultimately had increased to an intermediate level (at 3.4 years after ‘time 0’), reflecting some successful correction of fitness costs of escape variants by compensatory mutants or by the selection of alternative, higher fitness escape mutants. In contrast, although in the case of RI088 the transmitted virus was not known, the lack of multiple Gag escape mutants in RI088 appeared to have little impact on VRC compared to wildtype NL4-3 (Fig. 4b). These data are consistent with previous findings that the HLA-B*27:05- and HLA-B*57:01-associated escape mutants in Gag reduce viral replicative capacity, but even a relatively disabled virus can drive rapid progression to AIDS in the absence of effective HIV-specific CD8+ T cell activity.

## Discussion

We here describe two HIV-infected adults who express both HLA-B*27:05 and HLA-B*57:01, the most protective HLA class molecules against HIV disease progression, one of whom is a typical long-term non-progressor, maintaining low-to-undetectable viral loads over more than a decade of follow up, whilst the other is a rapid progressor, whose CD4+ T cell decline from immune control of HIV to AIDS occurs within a period of 3 years. In the rapid progressor studied here, we have shown using ultra-deep sequencing that escape mutations were selected in early infection across several of the known HLA-B*27:05 and HLA-B*57:01 epitopes, from the earliest timepoint when the CD4+ T cell count was 950 cells/mm^3^ and viral load was 65 copies/ml, preceding loss of immune control. However, the most dramatic viral sequence changes observed in this subject, coincident with rapid progression to AIDS, were associated with superinfection with a virus whose ancestry is substantially different from the original transmitted strain of HIV. Although many of the classical HLA-B*27:05 and HLA-B*57:01 ‘footprints’ [22] were present both pre- and post-superinfection, these commonly arise predictably and independently in individuals expressing these HLA molecules, and > 96% of amino acid changes were unrelated to CTL-driven escape.

In contrast, very little viral sequence change occurred over more than a decade in the non-progressor. Clonal sequencing reveals that escape mutants were generated all of this time, but in the majority of instances these were not selected. Finally, analysis of the viral replicative capacity (VRC) of the viruses in the two subjects revealed that differential rates of progression were not explained by lower VRC in the non-progressor. On the contrary, rapid progression occurred despite reductions in VRC in the subject with progressive disease.

Approximately 16% of Caucasians express HLA-B*27:05 and/or HLA-B*57:01 and ~ 60% of Caucasian elite controllers—approximately 0.3% of infected individuals overall [18]—express one or the other of these alleles [18]. Therefore, only a minority of individuals expressing HLA-B*27:05 and/or HLA-B*57:01—approximately 2%—become elite controllers, for reasons which remain unclear. Factors proposed to explain differential outcome in subjects with matched HLA-B allele expression may be categorised as immunological, genetic and virological. Immunological factors include breadth of the Gag-specific CTL response [3], avidity of the T cell receptor for the peptide-MHC complex [32], and ability of the TCR repertoire to recognise potential escape variants [33]. Genetic factors include the HLA-A and HLA-C [34, 35] and KIR alleles expressed [36], and the presence of CCR5 variants such as the delta-32 mutant [37]. Virological factors include replicative capacity of the transmitted virus and pre-adaptation of the transmitted virus to the HLA class I molecules expressed in the transmission recipient [38–41].

A further influence on HIV disease outcome is the introduction of co-infection with another blood-borne virus. HCV may skew the immunological response to HIV, for example by increasing immune activation [42]. The immunological influence of HCV therapy on HIV is complex, as exemplified by the APRICOT study in which HCV/HIV co-infected patients receiving IFNα had a 1.0 log reduction in HIV viral load, but also a reduction in absolute CD4+ T cell counts [43]. In the patient we describe here, it is likely that intercurrent acute HCV infection (and/or IFN-based therapy) may have contributed to HIV progression. However, this co-infection cannot explain the whole picture as changes in CD4+ T cell count and HIV viral load that herald progression to AIDS were already evident before the HCV infection occurred, and also the extent of the sequence changes observed can only be explained by HIV superinfection.

 In the case of the rapid progressor, the observation that several escape mutations were selected very early in the course of infection (prior to ‘time 0’) illustrates that the presence of a broad HIV-specific immune response does not provide complete protection against escape. Indeed, at the initial timepoint, when the CD4+ T cell count was 950 cells/mm^3^ and the HIV viral load was 65 copies/ml, five CTL responses were detectable (Fig. 3). Perhaps more significant is the observation of variants being generated in viral sequences derived from the non-progressor RI088 that did not get selected. Taken together with the data showing the relatively high viral replicative capacity of virus derived from RI088 compared to the R097 viruses, these findings are consistent with the quality of individual key HIV-specific responses being critical to outcome. These two case reports cannot provide definitive information on which specificities are most important to immune control, but are consistent with numerous previous studies indicating that the HLA-B*27:05-KK10 response plays a vital role in immune control of HIV in subjects expressing HLA-B*27:05.

The influence of genetic factors other than the HLA-B type has not been evaluated in these two study subjects. Although HLA-A and HLA-C alleles can influence immune control of HIV, HLA-B alleles have a greater impact than HLA-A or HLA-C [1, 35, 44]. The HLA-A alleles expressed by the progressor (A*11:01/24:02) and non-progressor (A*01:01/03:01) have not been associated with differential disease outcome and the HLA-C alleles expressed (C*02:02/06:02) were the same in both subjects.

 The changes in viral replicative capacity over time in R097 are of interest, showing an initial significant decrease in VRC as the B27/57 escape mutants are selected, and then a partial recovery towards the VRC of the original infecting strain from R096. These findings are consistent with many studies that have shown the ability of the virus to limit the cost of escape mutants either via the selection of alternative escape mutants [28] and/or the co-selection of compensatory mutants [27, 30, 31, 45–50]. However, although the VRC returns towards normal, it remains well below that of the original transmitted virus. In contrast, the VRC of the virus in the elite controller remains very close to the reference NL4-3 despite more than a decade of strong selection pressure on the virus.

## Conclusions

The description here of two HIV-infected subjects expressing both HLA-B*27:05 and HLA-B*57:01 with differential disease outcomes demonstrates the rapidity with which progression can occur, even when HIV-specific CTL responses restricted by these protective alleles are present. In this report rapid progression occurred following HIV superinfection. However, only a small minority of subjects expressing protective HLA alleles remain non-progressors. Consistent with previous studies, qualitative aspects of the HIV-specific CTL response are likely to play a critical part in maintaining long-term immune control of HIV infection.

## Additional files

**Additional file 1: Table 1.** The parameters used for the curves shown in Figure 4, together with the statistics for the overall fit and for the separate parameters.

**Additional file 2: Fig. 1.** Phylogenetic analysis of clonal HIV sequences from RI088. Maximum likelihood phylogenetic tree of 1091bp alignment of clonal DNA sequences across the Gag p17 and p24 genes. RI088 sequences are shown in colour (0.6 years in purple, 1.2 years in pink, 1.4 years in green, 3.4 years in orange, 10.8 years in blue, 11.3 years in red). 143 B B clade reference sequences from the US and UK collected between 2003 and 2011 from the Los Alamos database (https://www.hiv.lanl.gov/) are shown in black. Bootstrap values based on 1000 bootstrap replicates are shown in italics.

## Abbreviations

AIDS: acquired immunodeficiency syndrome
ART: antiretroviral therapy
CTL: cytotoxic T lymphocyte
DNA: deoxyribonucleic acid
ELISpot: enzyme-linked immunosorbent spot assay
Gag: group-specific antigen (capsid protein)
HCV: hepatitis C virus
HIV: human immunodeficiency virus
HLA: human leukocyte antigen
IFN: interferon
LOD: limit of detection
MHC: major histocompatibility complex
MSM: men who have sex with men
Nef: negative regulatory factor protein
PCR: polymerase chain reaction
RNA: ribonucleic acid
RT: reverse transcription
VRC: viral replicative capacity
