# Supplementary material for: Rapid HIV disease progression following superinfection in an HLA-B*27:05/B*57:01-positive transmission recipient
Source: Retrovirology. 2018 Jan 16;15:7. doi: 10.1186/s12977-018-0390-9 (PMC5771019; doi:10.1186/s12977-018-0390-9)
Supplement: Supplementary file 1 — Additional file 1: Table 1. The parameters used for the curves shown in Figure 4, together with the statistics for the overall fit and for the separate parameters. [file 12977_2018_390_MOESM1_ESM.pptx]

## Slide 1
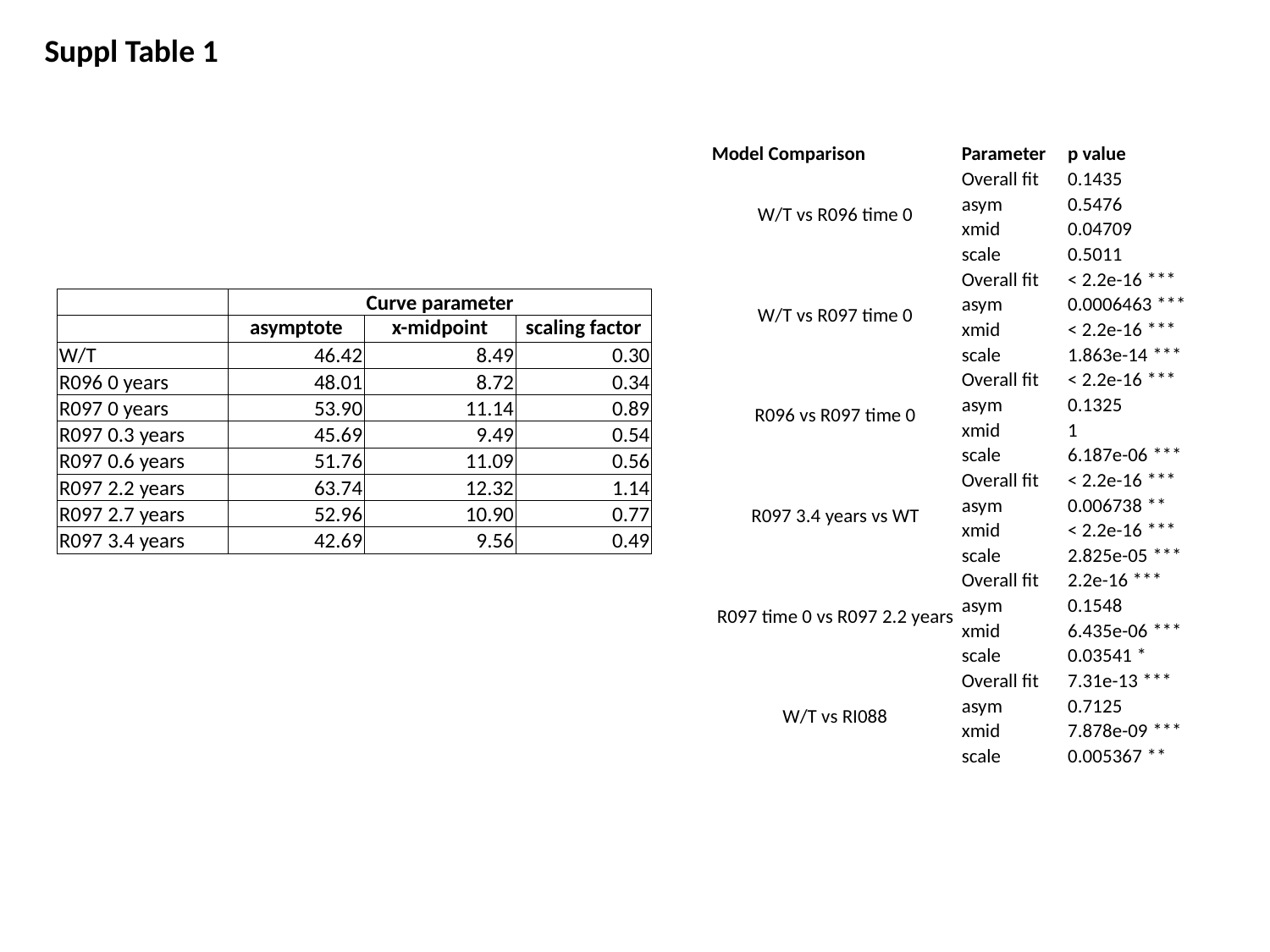

Suppl Table 1
| Model Comparison | Parameter | p value |
| --- | --- | --- |
| W/T vs R096 time 0 | Overall fit | 0.1435 |
| | asym | 0.5476 |
| | xmid | 0.04709 |
| | scale | 0.5011 |
| W/T vs R097 time 0 | Overall fit | < 2.2e-16 \*\*\* |
| | asym | 0.0006463 \*\*\* |
| | xmid | < 2.2e-16 \*\*\* |
| | scale | 1.863e-14 \*\*\* |
| R096 vs R097 time 0 | Overall fit | < 2.2e-16 \*\*\* |
| | asym | 0.1325 |
| | xmid | 1 |
| | scale | 6.187e-06 \*\*\* |
| R097 3.4 years vs WT | Overall fit | < 2.2e-16 \*\*\* |
| | asym | 0.006738 \*\* |
| | xmid | < 2.2e-16 \*\*\* |
| | scale | 2.825e-05 \*\*\* |
| R097 time 0 vs R097 2.2 years | Overall fit | 2.2e-16 \*\*\* |
| | asym | 0.1548 |
| | xmid | 6.435e-06 \*\*\* |
| | scale | 0.03541 \* |
| W/T vs RI088 | Overall fit | 7.31e-13 \*\*\* |
| | asym | 0.7125 |
| | xmid | 7.878e-09 \*\*\* |
| | scale | 0.005367 \*\* |
| | Curve parameter | | |
| --- | --- | --- | --- |
| | asymptote | x-midpoint | scaling factor |
| W/T | 46.42 | 8.49 | 0.30 |
| R096 0 years | 48.01 | 8.72 | 0.34 |
| R097 0 years | 53.90 | 11.14 | 0.89 |
| R097 0.3 years | 45.69 | 9.49 | 0.54 |
| R097 0.6 years | 51.76 | 11.09 | 0.56 |
| R097 2.2 years | 63.74 | 12.32 | 1.14 |
| R097 2.7 years | 52.96 | 10.90 | 0.77 |
| R097 3.4 years | 42.69 | 9.56 | 0.49 |
