# Supplementary figures and images for: Rapid HIV disease progression following superinfection in an HLA-B*27:05/B*57:01-positive transmission recipient
Source: Retrovirology. 2018 Jan 16;15:7. doi: 10.1186/s12977-018-0390-9 (PMC5771019; doi:10.1186/s12977-018-0390-9)

## Slide 1
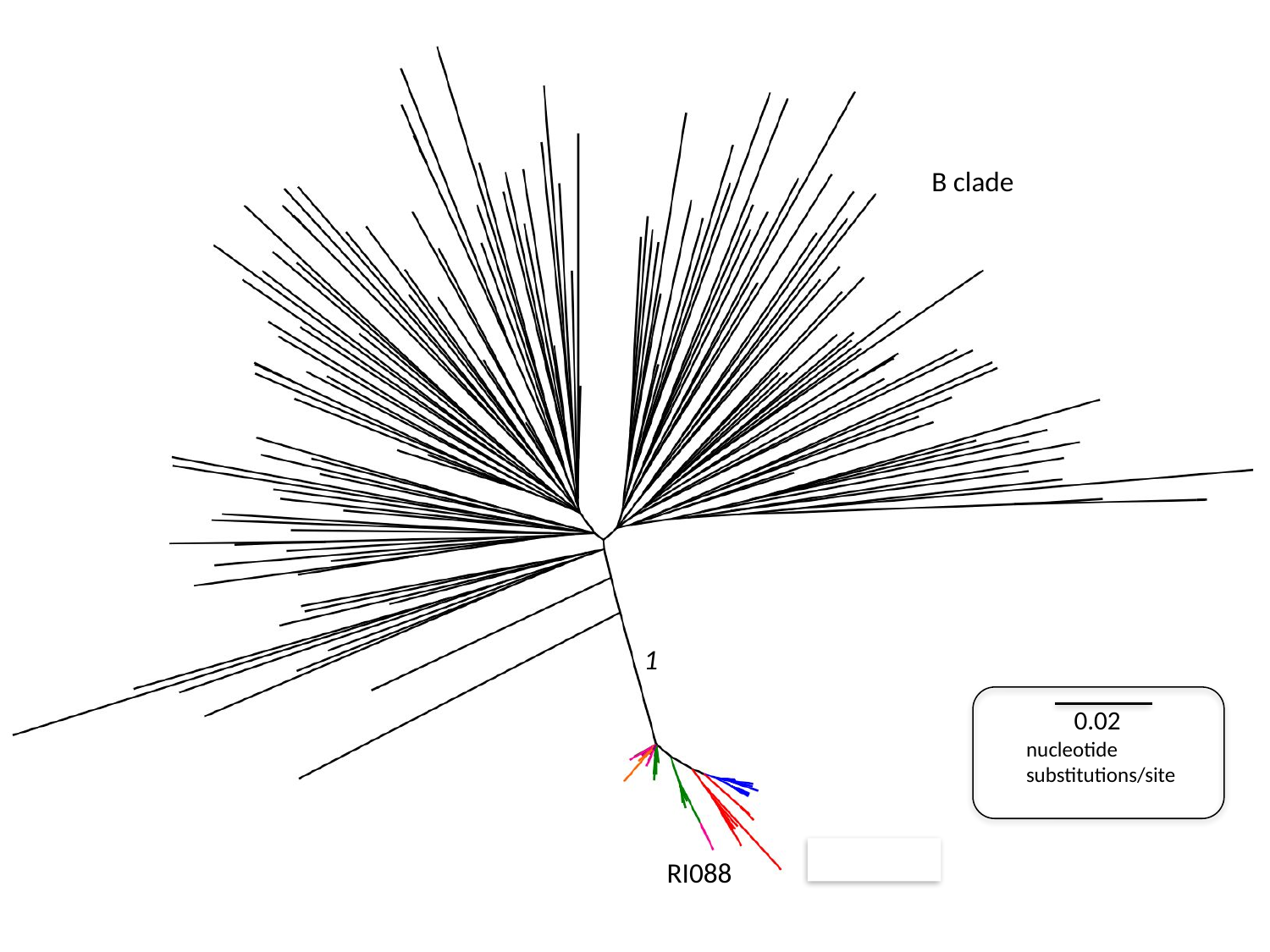

B clade
1
0.02
nucleotide
substitutions/site
RI088

Supplement: Supplementary file 2 — Additional file 2: Fig. 1. Phylogenetic analysis of clonal HIV sequences from RI088. Maximum likelihood phylogenetic tree of 1091bp alignment of clonal DNA sequences across the Gag p17 and p24 genes. RI088 sequences are shown in colour (0.6 years in purple, 1.2 years in pink, 1.4 years in green, 3.4 years in orange, 10.8 years in blue, 11.3 years in red). 143 B B clade reference sequences from the US and UK collected between 2003 and 2011 from the Los Alamos database (https://www.hiv.lanl.gov/) are shown in black. Bootstrap values based on 1000 bootstrap replicates are shown in italics. [file 12977_2018_390_MOESM2_ESM.pptx]
